# Poisoning by Green Wall Paper

**Published:** 1859-04

**Authors:** 


*Poisoning by Green Wall Paper.*—Our readers will recollect that some time ago, we called attention to the fact that slow arsenical poisoning had taken place, in consequence of the use of wall paper, which was colored by the arsenites of copper, Scheele's and Brunswick, or Paris green, as they are called in commerce. The sale of these papers having been somewhat interfered with, by the general agitation of the question of their insalubrity, attempts were made by the manufacturers to prove that they were really innocuous, and several chemical opinions were obtained sustaining this view of the matter.

Recently, however, the question has been revived in England. The distinguished toxicologist, Dr. Alfred Taylor, has called attention to the fact that the fine dust that settles on articles of furniture, in rooms papered with this beautiful, but dangerous variety of hangings, contains arsenic in restable quantity. His letter has brought out others from medical men, giving accounts of various forms of disease, chiefly gastro-intestinal derangements, occurring among the occupants of rooms so papered. The letters alluded to, have appeared in the columns of the London Medical Times and Gazette, and no candid reader of them can fail to be convinced of the great danger to health, if not to life, arising from the use of this paper.

